# A fatty acid analogue targeting mitochondria exerts a plasma triacylglycerol lowering effect in rats with impaired carnitine biosynthesis

**DOI:** 10.1371/journal.pone.0194978

**Published:** 2018-03-28

**Authors:** Carine Lindquist, Bodil Bjørndal, Christine Renate Rossmann, Asbjørn Svardal, Seth Hallström, Rolf Kristian Berge

**Affiliations:** 1 Department of Clinical Science, University of Bergen, Bergen, Norway; 2 Department of Heart Disease, Haukeland University Hospital, Bergen, Norway; 3 Institute of Physiological Chemistry, Medical University of Graz, Graz, Austria; Universite du Quebec a Montreal, CANADA

## Abstract

L-carnitine is important for the catabolism of long-chain fatty acids in the mitochondria. We investigated how the triacylglycerol (TAG)-lowering drug 2-(tridec-12-yn-1-ylthio)acetic acid (1-triple TTA) influenced lipid metabolism in carnitine-depleted, 3-(2,2,2-trimethylhydrazinium)propionate dehydrate (Mildronate; meldonium)-treated male Wistar rats. As indicated, carnitine biosynthesis was impaired by Mildronate. However, TAG levels of both plasma and liver were decreased by 1-triple TTA in Mildronate-treated animals. This was accompanied by increased gene expression of proteins involved in mitochondrial activity and proliferation and reduced mRNA levels of *Dgat2*, *ApoB* and *ApoCIII* in liver. The hepatic energy state was reduced in the group of Mildronate and 1-triple TTA as reflected by increased AMP/ATP ratio, reduced energy charge and induced gene expression of uncoupling proteins 2 and 3. The increase in mitochondrial fatty acid oxidation was observed despite low plasma carnitine levels, and was linked to strongly induced gene expression of carnitine acetyltransferase, translocase and carnitine transporter, suggesting an efficient carnitine turnover. The present data suggest that the plasma TAG-lowering effect of 1-triple TTA in Mildronate-treated rats is not only due to increased mitochondrial fatty acid oxidation reflected by increased mitochondrial biogenesis, but also to changes in plasma clearance and reduced TAG biosynthesis.

## Introduction

One of the risk factors characterizing the metabolic syndrome is dyslipidemia, including increased plasma triacylglycerol (TAG) [1]. Metabolic syndrome is associated with increased cardiovascular risk [2–5], in which cardiovascular disease is the main cause of death worldwide [6]. Non-alcoholic fatty liver disease is defined as accumulation of lipids in the liver, also associated with dyslipidemia [7], and is discussed to either be the hepatic manifestation of metabolic syndrome or an independent cardiovascular risk factor [8]. Between 20–40% of the general population in Western countries have non-alcoholic fatty liver disease [9]; however, no approved treatments have been defined besides alteration in diet and increased exercise. There are several events causing excessive intrahepatic accumulation of lipids such as increased lipid uptake by the liver, increased lipogenesis, and impaired synthesis and/or secretion of very-long density lipoprotein (VLDL) particles, preventing the removal of TAGs from the liver [10, 11]. Non-alcoholic fatty liver disease is referred to as a mitochondrial disease, including dysfunctional mitochondrial fatty acid oxidation and biogenesis [11, 12], thus drugs that target mitochondrial function can potentially improve both fatty liver disease and plasma TAG.

Carnitine is a conditionally essential molecule, and is mainly obtained through the diet in omnivorous humans [13, 14]. The endogenously carnitine biosynthesis from N^ε^-trimethyllysine is composed of four enzymatic steps catalyzed by N^ε^-trimethyllysine hydroxylase, 3-hydroxy- N^ε^-trimethyllysine aldolase, 4-N-trimethylaminobutyaldehyde dehydrogenase and γ-butyrobetaine hydroxylase. Carnitine biosynthesis is restricted to liver, kidney and brain [15], where it is either used directly or released into the circulation [14]. When transported, carnitine is taken up into cells through organic cation/carnitine transporters (OCTN) [16, 17]; thus, in healthy subjects, carnitine is maintained in a systemic homeostasis. Carnitine is crucial for mitochondrial β-oxidation, where long-chain fatty acyl-CoA needs to be converted to fatty acyl-carnitine by carnitine palmitoyltransferase I (CPT1) for transport over the outer mitochondrial membrane, and across the inner membrane through the carnitine-acylcarnitine translocase (CACT). Once inside the mitochondrial matrix, fatty acyl-carnitine is re-converted to fatty acyl-CoA by CPT2, ready for β-oxidation. As carnitines are important for mitochondrial β-oxidation, disturbance in carnitine transport can disturb fatty acid oxidation [18]. Carnitine is also important to maintain the pool of free CoA; surplus acyl-CoA including acetyl-CoA (i.e. the final product of β-oxidation) can reversibly be converted to acyl/acetylcarnitine by carnitine o-acetyltransferase, and thereafter be transported to the cytosol through CACT [14]. Treatment of carnitine deficiency involves large oral doses of carnitine to normalize β-oxidation, but this is not sufficient to normalize tissue carnitine concentrations [19, 20].

3-(2,2,2-trimethylhydrazinium)propionate dehydrate (meldonium; brand name Mildronate), is an analogue to the carnitine precursor γ-butyrobetaine, thus inhibiting γ-butyrobetaine hydroxylase, suppressing carnitine biosynthesis, leading to decreased carnitine concentration [21, 22]. As this mechanism stimulates glucose metabolism in the heart, it is used as a cardioprotective drug [23]. However, the decrease of hepatic carnitine levels has been shown to give development of fatty liver in animals [22, 24]. Thus, Mildronate-treated rats can be used as a model for carnitine depletion [22].

2-(tridec-12-yn-1-ylthio)acetic acid (C_15_H_26_O_2_S; 1-triple TTA) has the same length as palmitic acid, in which the β-carbon is substituted with a sulphur atom. In addition, it has a triple bond at the ω-end making it resistant to both β-oxidation and ω-degradation. 1-triple TTA was introduced by Lindquist et al. [25] as a mitochondrial activator and proliferator in rats. It was demonstrated to increase the hepatic mitochondrial fatty acid oxidation based on *in vitro* palmitoyl-CoA oxidation assay, enzyme activity assays and gene expression of enzymes involved in mitochondrial β-oxidation and the carnitine shuttle. It also markedly lowered TAG content in plasma and liver, while decreasing hepatic ATP levels. We also measured plasma carnitine levels where L-carnitine, acylcarnitines and the carnitine precursor γ-butyrobetaine was decreased evidently compared to control. Hence, we postulated that 1-triple TTA might be a potential treatment of overweight and fatty liver.

As 1-triple TTA was shown to increase mitochondrial activity and lipid catabolism in liver, lower TAG levels and carnitine levels in plasma, the aim of the present study was to investigate whether 1-triple TTA could beneficially influence lipid metabolism when carnitine biosynthesis is impaired.

## Materials and methods

### Animal study

The animal study was conducted according to the Guidelines for the Care and Use of Experimental Animals, and in accordance with the Norwegian legislation and regulations governing experiments using live animals. The Norwegian State Board of Biological Experiments with Living Animals approved the protocol (Permit number 2015–7367). All efforts were made to optimize the animal environment, and minimize suffering. Male Wistar rats, *Rattus Norvegicus*, 5 weeks old, were purchased from Taconic (Ejby, Denmark). Upon arrival the rats were randomized using Research Randomizer [26], labeled and placed in open cages, four in each cage, where they were allowed to acclimatize to their surroundings for one week. During the acclimatization and experiment period, the rats had unrestricted access to chow and tap water. The rats were kept in a 12 hours light/dark cycle at a constant temperature (22 ± 2°C) and a relative humidity of 55% (± 5%). Upon start of the experiment, the rats were block randomized to their respective interventions (31). During the three weeks long experiment, there were two rats in each cage separated with a divider that let them have sniffing contact. All three groups were given chow throughout the experiment. In addition, group 1, control (n = 8) received 0.5 mL 0.5% methylcellulose from Hospital Pharmacy (Bergen, Norway) daily. Group 2 (n = 6) received 0.11 g (550 mg/kg body weight) Mildronate ([3-(2,2,2-trimethylhydrazinium)propionate dehydrate], obtained from JSC Grindeks, Latvia) dissolved in 0.5 mL 0.5% methylcellulose daily. Group 3 (n = 6) received 0.11 g Mildronate and 0.02 g (100 mg/kg body weight) 1-triple TTA ([2-(tridec-12-yn-1-ylthio)acetic acid (C_15_H_26_O_2_S), obtained from Synthetica AS, Oslo, Norway) dissolved in 0.5 mL 0.5% methylcellulose daily. Mildronate dose was based on previous publications [27]. 1-triple TTA dose was determined by unpublished cell culture studies. Methylcellulose was given orally by gavage. All animals were weighed daily and feed intake was determined weekly. For euthanizing, rats were anaesthetized by inhalation of 5% isoflurane (Schering-Plough, Kent, UK), before cardiac puncture, after opening the abdomen in the midline. EDTA-blood was collected and immediately chilled on ice. The samples were centrifuged and plasma was stored at– 80°C prior to analysis. Liver was collected and weighed and a fresh sample from each liver was removed for β-oxidation analysis. The remaining part of the liver was immediately snap-frozen in liquid nitrogen and stored at– 80°C until further analysis.

### Quantification of plasma and liver parameters

#### Lipid analysis

Liver lipids were extracted from frozen samples according to Bligh and Dyer [28], evaporated under nitrogen, and re-dissolved in isopropanol before analysis. Lipids from liver and plasma were measured enzymatically on a Hitachi 917 system (Roche Diagnostics GmbH, Mannheim, Germany) using the triacylglycerol (Triglycerides GPO-PAP, 11730711) kit from Roche Diagnostics and phospholipid kit (Phospholipids FS, Ref 157419910930) from DiaSys (Diagnostic Systems GmbH, Holzheim, Germany).

#### Plasma carnitine and carnitine precursors

L-carnitine, trimethyllysine, γ-butyrobetaine, and acetylcarnitine were analyzed in plasma by high-performance liquid chromatography-MS/MS as described by Vernez et al. [29] with some modifications [30].

#### Hepatic high-energy phosphates

Freeze-clamped biopsies were taken from the liver and stored in liquid nitrogen. The sample preparation and high-performance liquid chromatography measurement of ATP, ADP and AMP were performed as previously described [31–33]. Details are given in Lindquist et al. [25]. Energy charge was calculated employing the following formula: Energy charge = (ATP + 0.5 ADP)/(AMP + ADP + ATP).

#### Hepatic enzyme assays

1 g fresh liver sample was chilled on ice and homogenized in 4 mL ice-cold sucrose medium (0.25 M sucrose, 10 mM HEPES, and 1 mM Na_4_EDTA, adjusted to a pH of 7.4 with KOH/HCl). The homogenates were centrifuged at 1030 RCF for 10 min at 4°C and the post-nuclear fraction was removed and used for further analysis [34]. Palmitoyl-CoA oxidation was measured immediately in the post-nuclear fraction from fresh liver as acid-soluble products, as described [35]. Further analyses were performed in frozen post-nuclear fraction: 3-ketothiolase (EC: 2.3.1.16) [36], Carnitine O-palmitoyltransferase 2 (CPT2, EC: 2.3.1.21) [37], citrate synthase (EC: 2.3.3.1) [38, 39], fatty acid synthase (FAS; EC: 2.3.1.85) [40], acetyl-CoA carboxylase (EC: 6.4.1.2) [41], citrate-ATP lyase (EC: 2.3.3.8) [42], acyl-CoA oxidase (ACOX; EC: 1.3.3.6) [43], glycerol-3-phosphate acyltransferase (GPAT; EC: 2.3.1.15) [44, 45], acyl-CoA synthetase (EC: 6.2.1.3) [46] and malonyl-CoA decarboxylase (EC: 4.1.1.9) [47]. The protein expression of carnitine/Acylcarnitine Translocase (CACT) was measured in frozen liver homogenized in PBS using ELISA kit from Nordic BioSite (Cat.: EKR1588; Täby, Sweden).

The protein concentration was measured by BioRad protein assay (Bio-Rad Laboratories, Hercules, CA, USA).

#### Hepatic gene expression analysis

Tissue samples (20 mg frozen liver) were homogenized in Rneasy Lysis Buffer from Qiagen (Cat.: 79216, Hilden, Germany) with 1% β-mercaptoethanol using Tissuelyser II (Qiagen) for 2x 2 min at 25 Hz, and total cellular RNA was further purified using the RNeasy mini kit (Qiagen, Hilden, Germany) including DNase digestion. 500 ng RNA was reverse transcribed using High Capacity cDNA Reverse Transcription Kits (Applied Biosystems, Waltham, Massachusetts, USA). qPCR was performed on Sarstedt 384-well Multiply-PCR plates (Sarstedt Inc., Newton, NC, USA) using ABI Prism 7900HT Sequence detection system from Applied Biosystems with the software SDS 2.3. Together with 2x Taqman buffer from Applied Biosystems, the following probes and primers from Applied Biosystems were used to detect mRNA levels of interests listed in alphabetic order: *Acaca* (Rn00573474_m1)–Acetyl-CoA carboxylase; *Acadm* (Rn00566390_m1)–Acyl-CoA dehydrogenase, medium chained; *Acadl* (Rn00563121_m1)–Acyl-CoA dehydrogenase, long chained; *Acadvl* (Rn00563649_m1)–Acyl-CoA dehydrogenase, very-long chained; *Acox1* (Rn01483784_m1)–Acyl-CoA Oxidase 1; *Aldh9a1* (Rn01491039_m1)– 4-N-trimethylaminobutyraldehyde dehydrogenase; *ApoB* (Rn01499054_m1)–Apolipoprotein B; *ApoCIII* (Rn00560743)–Apolipoprotein CIII; *Atp5c1* (Rn01487287_m1)–ATP synthase H^+^ transporting; mitochondrial F_1_ complex, gamma polypeptide 1; *Bbox1* (Rn00575255_m1)– γ-butyrobetaine hydroxylase; *Cd36* (Rn00580728_m1)–Cluster of differentiation 36, CD36; *Cpt1a* (Rn00580702_m1)–Carnitine palmitoyltransferase 1a; *Cpt2* (Rn00563995_m1)–Carnitine palmitoyltransferase 2; *Crat* (Rn01758585_m1)–Carnitine o-acetyltransferase; *Cycs* (Rn00820639_g1)–Cytochrome c, somatic; *Fabp1* (Rn00664587_m1)–Fatty acid binding protein 1; *Hadha* (Rn00590828_m1)–Hydroxyacyl-CoA dehydrogenase/3-ketoacyl-CoA thiolase/Enoyl-CoA hydratase (Trifunctional protein), alpha subunit; *Hadhb* (Rn00592435_m1)–Hydroxyacyl-CoA dehydrogenase/3-ketoacyl-CoA thiolase/Enoyl-CoA hydratase (Trifunctional protein), beta subunit; *Ndufa9* (Rn01462923_m1)–NADH dehydrogenase (Ubiquinone) 1 alpha subcomplex, 9; *Ppargc1a* (Rn00580241_m1)–Peroxisome proliferator-activated receptor gamma, coactivator 1; *Slc22a5* (Rn01471177_m1)–Solute carrier family 22 (Organic cation/Carnitine transporter), member 5; *Slc25a20* (Rn00588652_m1)–Solute carrier family 25 (Carnitine/Acylcarnitine Translocase), Member 20; *Tfam* (Rn00580051_m1)–Transcription factor A, mitochondrial; *Tmlhe* (Rn00591314_m1)–N^ε^-trimethyllysine hydroxylase; *Ucp2* (Rn01754856_m1)–Uncoupling protein 2; *Ucp3* (Rn00565874_m1)–Uncoupling protein 3; *Vldlr* (Rn00565784_m1)–Very low-density lipoprotein receptor. Each probe was run with standard curve where either a sample or URRR was used. Results were normalized to *Rplp0* and then set as relative to control. 18S was used as positive control.

### Statistical analysis

One-way ANOVA was performed, and in the cases of statistically significance (p<0.05), Fisher Least Significance Test (LSD) was conducted to determine which groups differed [48]. P<0.05 was considered statistically significant, but p-values above 0.01 are defined in text. The results are shown as mean ± standard deviation (SD) of 6–8 rats per group. mRNA levels were normalized to the house keeping gene *Rplp0* and set as relative to controls. Pearson’s correlation coefficients were used when comparing two independent variables. The statistics was performed using IBM SPSS Statistics for Windows, Version 22.0 (IBM Corp. Armonk, USA) and graphs were designed with GraphPad Prism for Windows, Version 6.00 (GraphPad Software, La Jolla, CA, USA, www.graphpad.com).

## Results

In this study, rats were given a daily dose of either Mildronate or Mildronate in combination with an artificial fatty acid 1-triple TTA, earlier shown to increase β-oxidation and mitochondrial proliferation. The Mildronate groups are compared to each other and to a control group. All rats were given chow through-out the three weeks long experiment. The feed intake and weight gain were similar in all groups, as were the organ weights of heart and epididymal WAT (Table 1). However, the liver index was significantly higher in rats treated with both Mildronate and 1-triple TTA compared to Mildronate and control group.

**Table 1:** Weights and feed intake of male Wistar rats after three weeks of treatment.

Weights	C	M	MT
	n = 8	n = 6	n = 6
	mean±S.D.	mean±S.D.	mean±S.D.
**Total weight gain [g]**	94 ± 14^a^	86 ± 7.4^a^	83 ± 14^a^
**Chow eaten total [g]**	502 ± 47^a^	496 ± 34^a^	511 ± 58^a^
**Epididymal WAT [g]**	4.2 ± 1.6^a^	3.7 ± 0.3^a^	3.6 ± 1.9^a^
**Liver index [%]**	3.5 ± 0.2^a^	3.8 ± 0.5^a^	5.1 ± 0.3^b^

Values are shown as mean ± SD (n = 6–8). One-Way ANOVA with p<0.05 was followed by Fisher LSD to determine significant difference between all three groups: Different letters above bars indicate significant difference between group mean values, p<0.05; same letters above bars indicate no significant difference between group mean values p>0.05. C–Control, M–Mildronate (550 mg/kg body weight), MT–combination of Mildronate (550 mg/kg body weight) and 1-triple TTA (100 mg/ kg body weight).

### The effect on plasma carnitine and carnitine derivatives

**Fig 1:**
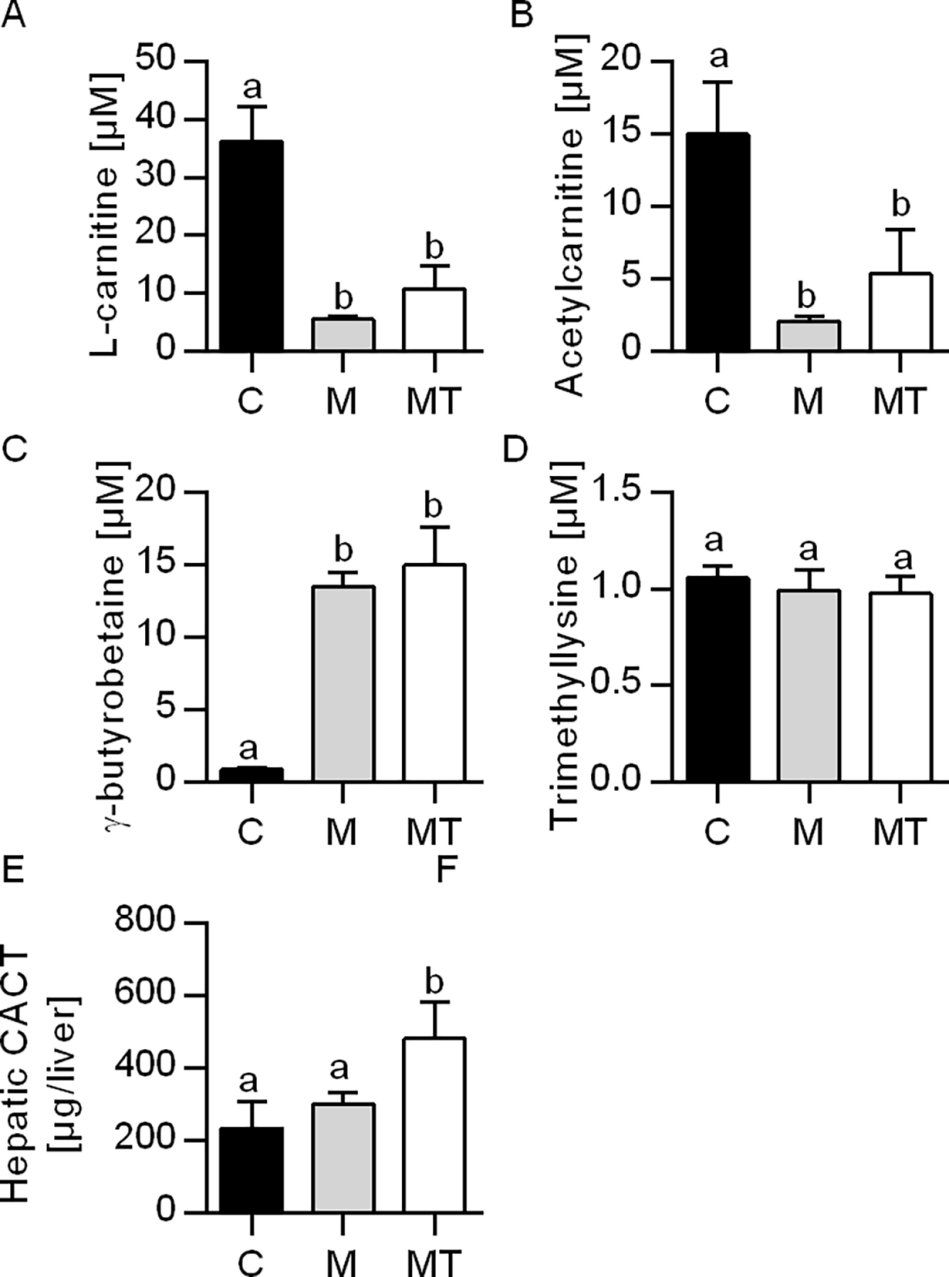
Plasma levels of carnitine derivatives and carnitine precursors in male Wistar rats after three weeks of treatment. **A.** Plasma L-carnitine; **B.** Plasma acetylcarnitine; **C.** Plasma γ-butyrobetaine; **D.** Plasma trimethyllysine; **E.** Protein expression of carnitine translocase (CACT). Values are shown as mean ± SD (n = 6–8). One-Way ANOVA with p<0.05 was followed by Fisher LSD to determine significant difference between all three groups. Different letters above bars indicate significant difference between group mean values, p<0.05; same letters above bars indicate no significant difference between group mean values p>0.05. C–Control, M–Mildronate (550 mg/kg body weight), MT–combination of Mildronate (550 mg/kg body weight) and 1-triple TTA (100 mg/ kg body weight).

In the present study, the plasma levels of L-carnitine (Fig 1A) and acetylcarnitine (Fig 1B) were reduced in the Mildronate-fed group (85 and 87% reduction, respectively) and in the combination treatment of Mildronate and 1-triple TTA (70 and 65% reduction, respectively) compared to control. This was followed by a 16-fold increase in plasma level of γ-byturobetaine in the two Mildronate-treated groups compared to controls (Fig 1C), while *Bbox1* mRNA showed a small, but significant reduction by Mildronate+1-triple TTA compared to control (p = 0.021), and to Mildronate (p = 0.003) (Table 2). Moreover, in both experimental groups, the plasma level of trimethyllysine (Fig 1D) and the mRNA levels of *Tmlhe* and *Aldh9a1* remained constant (Table 2). The gene expression of the carnitine transporter OCTN2 (*Slc22a5*) was induced 7-fold by 1-triple TTA in Mildronate-treated rats compared to both control and Mildronate (Table 2). The gene expression of hepatic carnitine o-acetyltransferase (*Crat*) was induced ~30-fold by 1-triple TTA in Mildronate-treated rats and no change of Mildronate compared to control. The gene (Table 2) and protein (Fig 1E) expression of CACT (*Slc25a20*) (Table 2) were induced by1-triple TTA in Mildronate-treated rats compared to control and Mildronate treatment.

**Table 2:** Hepatic gene expression in Wistar male rats after three weeks of treatment.

Gene symbol	C	M	MT	Gene Symbol	C	M	MT
	n = 8	n = 6	n = 6		n = 8	n = 6	n = 6
	mean±SD.	mean±SD.	mean±SD.		mean±SD.	mean±SD.	mean±SD.
**Carnitine synthesis**				**Mitochondrial biogenesis**			
*Tmlhe*	1.0 ±0.1^a^	0.9 ±0.1^a^	0.9 ±0.1^a^	*Tfam*	1.0 ±0.1^a^	1.1 ±0.1^a^	1.3 ±0.1^b^
*Aldh9a1*	1.0 ±0.1^a^	1.1 ±0.1^a^	0.9 ±0.2^a^	*Ppargc1a*	1.0 ±0.3^a^	0.6 ±0.2^b^	1.2 ±0.2^a^
*Bbox1*	1.0 ±0.1^a^	1.1 ±0.1^a^	0.8 ±0.2^b^	**Mitochondrial respiration**			
**Carnitine transporter**				*Ndufa9*	1.0 ±0.1^a^	1.0 ±0.1^a^	1.2 ±0.1^b^
*Slc22a5*	1.0 ±0.2^a^	0.9 ±0.2^a^	7.4 ±2.3^b^	*Cycs*	1.0 ±0.1^a^	1.1 ±0.1^a^	1.4 ±0.1^b^
*Slc25a20*	1.0 ±0.1^a^	1.0 ±0.1^a^	3.0 ±0.4^b^	*Atp5c1*	1.0 ±0.1^a^	1.1 ±0.0^a^	1.3 ±0.1^b^
**Carnitine transferases**				*Ucp2*	1.0 ±0.1^a^	0.9 ±0.1^a^	1.5 ±0.6^b^
*Cpt1a*	1.0 ±0.3^a^	0.8 ±0.3^a^	2.6 ±0.8^b^	*Ucp3*	0.0 ±0.0^a^	0.0 ±0.0^a^	15 ±12^b^
*Cpt2*	1.0 ±0.2^a^	0.8 ±0.1^a^	3.3 ±0.6^b^	**Lipogenesis**			
*Crat*	1.0 ±0.3^a^	1.2 ±0.4^a^	27 ±7.2^b^	*Acaca*	1.0 ±0.3^a^	0.7 ±0.4^a^	0.8 ±0.2^a^
**β-oxidation**				*Fasn*	1.0 ±0.5^a^	1.0 ±0.9^a^	0.7 ±0.3^a^
*Acadl*	1.0 ±0.1^a^	1.0 ±0.1^a^	2.4 ±0.2^b^	*Dgat1*	1.0 ±0.1^a^	1.0 ±0.0^a^	1.6 ±0.2^b^
*Acadm*	1.0 ±0.1^a^	1.0 ±0.1^a^	2.2 ±0.3^b^	*Dgat2*	1.0 ±0.1^a^	0.9 ±0.1^a^	0.5 ±0.2^b^
*Acadvl*	1.0 ±0.0^a^	1.0 ±0.1^a^	2.1 ±0.2^b^	**Fatty acid uptake**			
*Hadha*	1.0 ±0.2^a^	1.4 ±0.3^a^	4.0 ±1.1^b^	*Cd36*	1.0 ±0.6^a^	0.7 ±0.1^a^	5.3 ±2.0^b^
*Hadhb*	1.0 ±0.1^a^	1.1 ±0.1^a^	4.9 ±0.7^b^	*Fabp1*	1.0 ±0.2^a^	0.9 ±0.1^a^	2.8 ±0.5^b^
*Acox1*	1.0 ±0.1^a^	1.0 ±0.4^a^	10 ±2.9^b^	**Lipid transport**			
**PPAR isoforms**				*ApoB*	1.0 ±0.2^a^	1.0 ±0.2^a^	0.7 ±0.1^b^
*Pparα*	1.0 ±0.3^a^	0.7 ±0.2^a^	1.1 ±0.4^a^	*ApoC-II*	1.0 ±0.1^a^	1.0 ±0.1^a^	0.7 ±0.2^b^
*Pparδ*	1.0 ±0.3^a^	2.7 ±2.0^b^	0.9 ±0.4^a^	*ApoC-III*	1.0 ±0.2^a^	0.9 ±0.2^a^	0.3 ±0.2^b^
*Pparγ*	1.0 ±0.3^a^	1.0 ±0.1^a^	1.0 ±0.2^a^	*Vldlr*	1.0 ±0.3^a^	1.0 ±0.3^a^	3.2 ±2.7^b^

All values were normalized to *Rplp0* and relative to control except for *Ucp3* which is only shown in comparison to *Rplp0*. Values are shown as mean ± SD (n = 6–8). One-Way ANOVA with p<0.05 was followed by Fisher LSD to determine significant difference between all three groups: Different letters above bars indicate significant difference between group mean values, p<0.05; same letters above bars indicate no significant difference between group mean values p>0.05. C–Control,–Mildronate (550 mg/kg body weight), MT–combination of Mildronate (550 mg/kg body weight) and 1-triple TTA (100 mg/ kg body weight).

Abbreviations: *Tmlhe*–N^ε^-trimethyllysine hydroxylase; *Aldh9a1*–4-N-trimethylaminobutyraldehyde dehydrogenase; *Bbox1* – γ-butyrobetaine hydroxylase; *Crat*–Carnitine o-acetyltransferase; *Slc22a5* –Organic cation/carnitine transporter; *Slc25a20* –Carnitine/Acylcarnitine translocase; *Cpt1a/2* –Carnitine palmitoyltransferase 1a/2, *Acad(l/m/vl)*–Acyl-CoA dehydrogenase (long chained/medium chained/very-long chained respectively); *Hadha* and *Hadhb*–Hydroxyacyl-CoA Dehydrogenase/3-Ketoacyl-CoA Thiolase/Enoyl-CoA Hydratase (Trifunctional Protein) alpha and beta subunit; *Pparα/δ/γ –*Peroxisome proliferator-activated receptor alpha/delta/gamma; *Ppargc1a* –Peroxisome proliferator-activated receptor gamma, coactivator 1; *Tfam*–Transcription factor A, mitochondrial;; *Ndufa9* –NADH dehydrogenase (Ubiquinone) 1 Alpha Subcomplex, 9, 39kDa; *Cycs*–Cytochrome C, somatic; *Atp5c1* –ATP synthase H^+^ Transporting, Mitochondrial F1 Complex, Gamma Polypeptide 1; *Ucp2/3* –Uncoupling protein 2/3; *Acaca–*Acetyl-CoA carboxylase; *Fasn–*Fatty acid synthase; *Dgat1/2* –Diacylglycerol O-acyltransferase 1/2; *Cd36* –Cluster of differentiation 36 (CD36), a fatty acid translocase; *Fabp1* –Fatty acid binding protein 1; *ApoB*–Apolipoprotein B; *ApoCII/III*–Apolipoprotein C-II/III; *Vldlr*–Very-low density lipoprotein receptor.

### The effect on fatty acid oxidation and mitochondrial function

**Fig 2:**
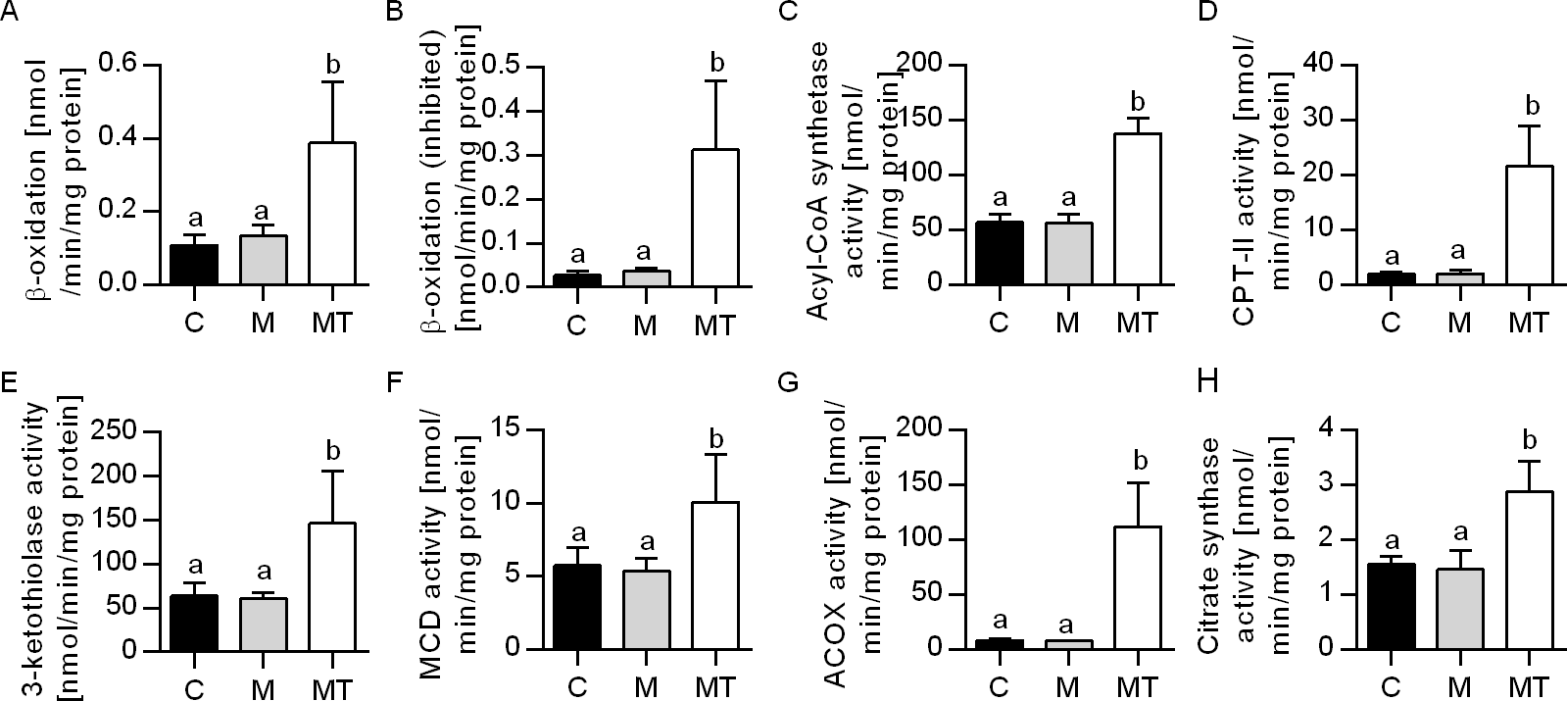
Hepatic β-oxidation and enzyme activities involved in lipid catabolism in male Wistar rats after three weeks of treatment. (A) Total β-oxidation of palmitoyl-CoA in liver; (B) Total β-oxidation of palmitoyl-CoA with addition of malonyl-CoA in liver; (C) Enzyme activity of acyl-CoA synthetase; (D) Enzyme activity of carnitine palmitoyltransferase (CPT) 2; (E) Enzyme activity of 3-ketothiolase; (F) Enzyme activity of malonyl-CoA decarboxylase (MCD); (G) Enzyme activity of acyl-CoA oxidase (ACOX); (H) Enzyme activity of citrate synthase. Values are shown as means ± SD (n = 6–8). One-Way ANOVA with p<0.05 was followed by Fisher LSD to determine significant difference between all three groups: Different letters above bars indicate significant difference between group mean values, p<0.05; same letters above bars indicate no significant difference between group mean values p>0.05. C–Control, M–Mildronate (550 mg/kg body weight), MT–combination of Mildronate (550 mg/kg body weight) and 1-triple TTA (100 mg/ kg body weight).

**Fig 3:**
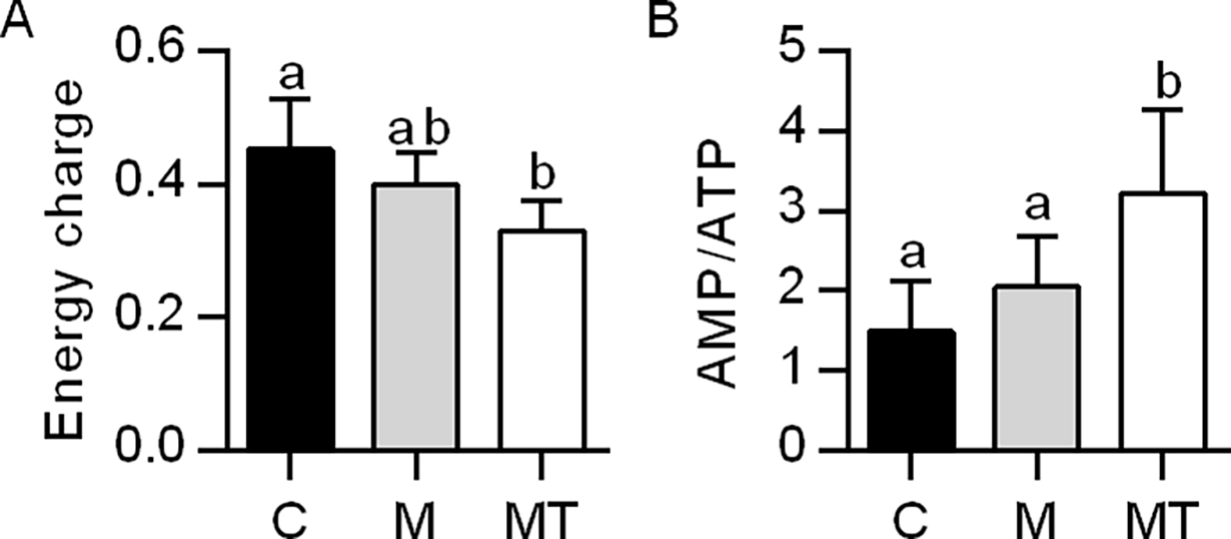
Hepatic energy parameters in male Wistar rats after three weeks of treatment. (A) Energy charge (ATP + 0.5 ADP)/(AMP + ADP + ATP). (B) Ratio of AMP and ATP. Values are shown as mean ± SD (n = 6–8). One-Way ANOVA with p<0.05 was followed by Fisher LSD to determine significant difference between all three groups: Different letters above bars indicate significant difference between group mean values, p<0.05; same letters above bars indicate no significant difference between group mean values p>0.05. C–Control, M–Mildronate (550 mg/kg body weight), MT–combination of Mildronate (550 mg/kg body weight) and 1-triple TTA (100 mg/ kg body weight).

The total palmitoyl-CoA β-oxidation in liver, both with and without inhibition by malonyl-CoA were increased by the combined intervention of Mildronate and 1-triple TTA compared to both control and Mildronate (Fig 2A and 2B). This was accompanied by increased expression of genes involved in mitochondrial β-oxidation (*Acadl*, *Acadm*, *Acadvl*, *Hadha*, *Hadhb*) and hepatic genes in the carnitine shuttle (*Cpt1a*, *Cpt2*) (Table 2), as well as enzyme activities of acyl-CoA synthetase, CPT2, 3-ketothiolase and malonyl-CoA decarboxylase (Fig 2C–2F). Moreover, the peroxisomal fatty acid oxidation seemed to be increased in this experimental group as the gene expression (Table 2) and activity (Fig 2G) of ACOX1 was strongly increased compared to both control and Mildronate without any alteration in the gene expression of PPAR isoforms (Table 2). Moreover, combination of Mildronate and 1-triple TTA increased hepatic genes in mitochondrial biogenesis and respiration, *Tfam*, *Cycs*, *Ndufa9*, *Atp5c1* (Table 2) as well as the activity of citrate synthase compared to control and Mildronate (Fig 2H). Finally, a possible partial uncoupling of mitochondria by 1-triple TTA resulted as the hepatic energy charge decreased (i.e. higher concentration of AMP) compared to control (p = 0.002) (Fig 3), and higher AMP/ATP ratio compared to control (p = 0.001) and Mildronate (p = 0.023). This was followed by a higher gene expression of UCP2 (p = 0.023, p = 0.017) and UCP3 (p<0.001, p<0.001) (Table 2) compared to control and Mildronate respectively. Noteworthy, Mildronate treatment alone seemed not to affect liver lipid metabolism, genes and enzymatic activities except for increased mRNA level of *Pparδ* compared to control (p = 0.012) and Mildronate+1-triple TTA (p = 0.011) and decreased mRNA level of *Ppargc1a* compared to both control (p = 0.019) and Mildronate+1-triple TTA (p = 0.001) (Table 2).

### The effect on hepatic TAG level and lipogenesis

**Fig 4:**
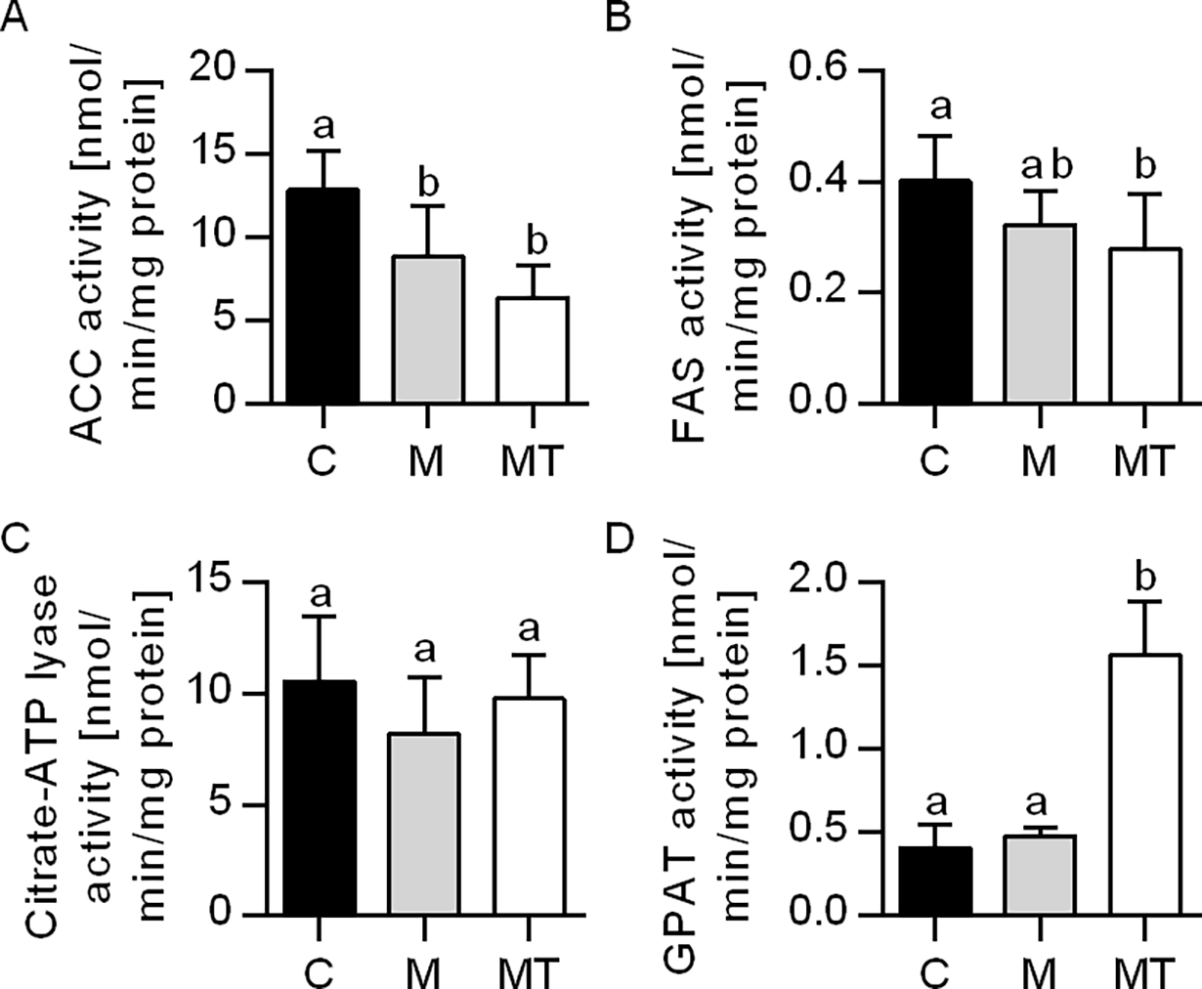
Hepatic enzyme activities involved in lipid anabolism in male Wistar rats after three weeks of treatment. (A) Enzyme activity of acetyl-CoA carboxylase (ACC). (B) Enzyme activity of fatty acid synthase (FAS). (C) Enzyme activity of citrate-ATP lyase. (D) Enzyme activity of glycerol-3-phosphate transferase (GPAT). Values are shown as mean± SD (n = 6–8). One-Way ANOVA with p<0.05 was followed by Fisher LSD to determine significant difference between all three groups: Different letters above bars indicate significant difference between group mean values, p<0.05; same letters above bars indicate no significant difference between group mean values p>0.05. C–Control, M–Mildronate (550 mg/kg body weight), MT–combination of Mildronate (550 mg/kg body weight) and 1-triple TTA (100 mg/ kg body weight).

The liver TAG content remained constant after Mildronate administration, whereas the hepatic level of TAG for the combination treatment of Mildronate and 1-triple TTA was significantly reduced compared to Mildronate and controls (Table 3). The hepatic mRNA levels of genes involved in import of fatty acids, *Cd36* and *Fabp1*, were increased in the combination group of Mildronate and 1-triple TTA (Table 3). As the availability of fatty acids is a major determinant of the hepatic TAG synthetic rate, it was important to investigate the effect of Mildronate and 1-triple TTA not only on fatty acid oxidation, but also on lipogenesis. Fig 4A–4C showed that treatment with Mildronate and combination of Mildronate and 1-triple TTA resulted in inhibited acetyl-CoA carboxylase activity. While the combination of Mildronate and 1-triple TTA also decreased FAS activity (p = 0.013), the citrate-ATP lyase activity remained constant in both experimental groups compared to control. Mildronate alone seemed to have no effect on the hepatic triacylglycerol biosynthesis, as the GPAT activity and mRNA levels of *Dgat1* and *Dgat2* were unchanged (Fig 4D) (Table 3). Interestingly, in the combination group of Mildronate and 1-triple TTA, the GPAT activity was stimulated and this was associated with upregulation of *Dgat1* mRNA and a higher hepatic phospholipid level (Table 3). Noteworthy, in this feeding group, the mRNA level of *Dgat2* was decreased (Table 3), associated with a negative correlation between hepatic TAG and *Dgat2* (Table 4).

**Table 3:** Plasma and liver lipids in male Wistar rats after three weeks of treatment.

Lipids	C	M	MT
	n = 8	n = 6	n = 6
	mean±SD	mean±SD	mean±SD
**Plasma TAG** **[mmol/L]**	1.8 ± 0.6^a^	1.6 ± 0.2^a^	0.6 ± 0.4^b^
**Liver TAG** **[μmol/gram liver]**	5.6 ± 1.3^a^	6.0 ± 0.8^a^	2.9 ± 2.1^b^
**Liver phospholipids** **[μmol/gram liver]**	25 ± 1^a^	24 ± 1^a^	27 ± 2^b^

Values are shown as mean ± SD (n = 6–8). One-Way ANOVA with p<0.05 was followed by Fisher LSD to determine significant difference between all three groups: Different letters above bars indicate significant difference between group mean values, p<0.05; same letters above bars indicate no significant difference between group mean values p>0.05. C–Control,–Mildronate (550 mg/kg body weight), MT–combination of Mildronate (550 mg/kg body weight) and 1-triple TTA (100 mg/ kg body weight).

**Table 4:** Correlations of TAG and hepatic β-oxidation and *Dgat2* mRNA in male Wistar rats after three weeks of treatment.

	TAG in plasma (n = 20)	TAG in liver (n = 20)
**β-oxidation (n = 20)**	r = - 0.720 ***	
***Dgat2* mRNA (n = 20)**	r = 0.499 *	r = 0.667 **

Pearson correlation coefficients (r) between β-oxidation and triacylglycerol (TAG) in liver and between β-oxidation and TAG in plasma.

*p≤0.05

***p≤0.01

***p≤0.001.

### Effect on plasma TAG level and lipoprotein gene expression

Mildronate treatment did not change the plasma TAG level compared to controls, but noteworthy, in the combination of Mildronate and 1-triple TTA, plasma TAG was significantly lowered (Table 2). Moreover, a negative correlation between plasma TAG and mitochondrial fatty acid β-oxidation as well as plasma TAG and *Dgat2* occurred (Table 3).

As changes in VLDL-TAG secretion rate and plasma clearance rate might contribute to the TAG-lowering effect, we examined whether the gene expression of *ApoB*, *ApoCII*, *ApoIII* and *Vldlr* (i.e. VLDL receptor) were changed after 1-triple TTA. Table 2 showed that the combination of Mildronate and 1-triple TTA downregulated the mRNA levels of *ApoB*, *ApoCII*, *ApoCIII* and *Vldlr* (p = 0.016, p = 0.021) compared to control and Mildronate, whereas Mildronate administration itself had no effect on these genes.

## Discussion

This work demonstrated that despite the substantial decrease in plasma carnitine in Mildronate-treated rats, 1-triple TTA was able to lower plasma and liver TAG concentrations. These improvements are most likely due to increased hepatic mitochondrial fatty acid oxidation, where carnitine is efficiently consumed, accompanied by reduced TAG synthesis and increased plasma clearance. These data were not associated with altered feed intake or weight gain.

Mildronate is associated with reduced plasma carnitine levels [49]. In the present study, Mildronate alone and combined with 1-triple TTA decreased plasma carnitine and acetylcarnitine levels compared to controls, accompanied by an increase in plasma γ-butyrobetaine levels. Noteworthy, Mildronate did not affect the hepatic gene expression of enzymes involved in carnitine biosynthesis. However, the combination of Mildronate and 1-triple TTA decreased *Bbox1* compared to Mildronate and to some extent control group (p = 0.021). 1-triple TTA treatment in rats has previously been shown to reduce *Bbox1* expression and to lower plasma carnitine and acetylcarnitine levels by 40–50%, but also γ-butyrobetaine by 40% [25]. This suggests different mechanisms: while Mildronate reduces carnitine levels by inhibiting the enzyme involved in carnitine biosynthesis [49], 1-triple TTA may affect gene expression of *Bbox1* and most likely increase carnitine consumption [25]. In the presence of Mildronate, 1-triple TTA showed no additional reduction of plasma carnitine compared to Mildronate (71% vs 85%), but a larger reduction than previously reported with 1-triple TTA alone (52%, Lindquist et al. [25]). Hence, Mildronate seemed to dominate the carnitine decrease in the combined intervention group.

The substantial lowering of plasma carnitine could prevent sufficient transport of long-chain fatty acids into the mitochondria, thus inhibiting fatty acid oxidation [21]. β-oxidation was unchanged by Mildronate compared to control, which might be due to the *in vitro* assay used, where carnitine was added to the reaction mix [50]. Mildronate also had no effect on the genes or activities of enzymes involved in hepatic mitochondrial or peroxisomal fatty acid oxidation. In contrast, the combination of Mildronate and 1-triple TTA increased total *in vitro* fatty acid oxidation, gene expression and enzyme activities related to both mitochondrial and peroxisomal β-oxidation, demonstrating an increased potential for fatty acid catabolism, associated with an increased liver index as earlier described [25]. Also, gene expression of *Ucp2* and *Ucp3* was increased by 1-triple TTA in both non-treated [25] and Mildronate-treated rats. The role of these proteins is not well-established. However, they have been linked to ROS degeneration and associated with increased β-oxidation [51–53]. The lowered energy charge and increased AMP/ATP ratio, combined with the previous observation that 1-triple TTA has shown not to activate AMPK [25], indicated that 1-triple TTA could lead to a partial uncoupling in Mildronate-treated rats. 1-triple TTA was earlier shown to increase mitochondrial biogenesis, based on mitochondrial DNA levels and electron microscopy [25]. The increased mitochondrial activity (activities and gene expression) and TFAM gene expression indicates that 1-triple TTA also increased mitochondrial biogenesis in Mildronate-treated as well as non-treated rats [25]. Although PGC-1α gene expression was not increased, we have postulated before that this might not be essential for increased biogenesis [25]. Based on these results, we also postulate that an increased number of mitochondria are responsible for the increased β-oxidation. In a situation with reduced availability of carnitine, the peroxisomes will still be able to catabolize long-chain fatty acids. No increase in peroxisomal ACOX activity was observed in the Mildronate group, but ACOX activity was strongly induced by Mildronate and 1-triple TTA co-treatment indicating increased peroxisomal activity. Increased peroxisomal fatty acid oxidation is also observed in 1-triple TTA without Mildronate (data not shown); hence, increased peroxisomal oxidation seems not to be due to impaired carnitine synthesis. In addition to the implied mitochondrial uncoupling, it should be emphasized that mitochondria are the far more quantitatively dominating organelles in liver cells [54], suggesting that the increase in mitochondrial fatty acid oxidation has a greater impact on the total fatty acid oxidation than peroxisomal fatty acid oxidation.

Moreover, GPAT activity in liver was increased in the combined intervention group, along with increased hepatic levels of phospholipids. This is in line with an increased need for intracellular membrane structures during mitochondrial proliferation. Hence, evidence points to an increased mitochondrial fatty acid oxidation despite carnitine depletion. Mildronate has previously been shown to decrease carnitine concentrations in liver and plasma, and increase urinary carnitine [22]. Hence, it would have been interesting to investigate the hepatic and urinary carnitine levels in this study. Unfortunately, we have not succeeded to do so, and hence we cannot determine if Mildronate treatment lowered hepatic carnitine levels. Mildronate was previously shown to be a substrate for OCTN2 [55], thus a competitive inhibitor [56], while also leading to increased mRNA levels of OCTN2 [22]. Although Mildronate did not alter hepatic gene expression of OCTN2 in this study (*Slc22a5*, Table 2), Mildronate may have still altered the activity of OCTN2. The gene and protein expression of the mitochondrial translocase CACT were unchanged by Mildronate compared to control. Mildronate has been shown to act as a competitive inhibitor of CACT [57], and thus could potentially inhibit the mitochondrial translocation of acylcarnitines. However, the inhibitory potency has been reviewed as weak [56]. Whether Mildronate changed the intramitochondrial carnitine level and thereby fatty acid metabolism through either reduced carnitine levels or inhibited CACT activity should be considered, but we do not have data to confirm this. 1-triple TTA was previously shown to increase OCTN2, CACT and acyltransferase gene expression (*Crat*) [25]. Here, 1-triple TTA increased the same genes, as well as the protein expression of CACT. Hence, it seems that 1-triple TTA efficiently imports carnitine to the liver and may increase its turnover, allowing a high level of long-chained fatty acid oxidation by 1-triple TTA in rats with altered carnitine biosynthesis, and thus lowering TAG in plasma and liver. Mildronate increased gene expression of hepatic *Pparδ*, which is important for the modulation of enzymes involved in gluconeogenesis as well as liver tissue recovery after chronic insult and lipotoxicity [58]. Whether this increase is of relevance should be considered.

The liver is important in regulation of plasma TAG, which is determined by a balance between hepatic TAG biosynthesis and secretion on one hand and plasma TAG clearance on the other. 1-triple TTA did not lead to epididymal WAT weight reduction, but this could be due to the short period of treatment. As discussed above, increased mitochondrial fatty acid oxidation is important for the TAG-lowering effect by 1-triple TTA. However, whether the observed reduction in plasma TAG during 1-triple TTA administration in the presence of Mildronate could also be influenced by reduced synthesis, reduced hepatic output, enhanced clearance or a combination of these factors, has to be further discussed: firstly, consistent with reduced lipogenesis, 1-triple TTA treatment of carnitine–depleted rats resulted in decreased activities of acetyl-CoA carboxylase and FAS, whereas the citrate-ATP lyase activity remained unchanged compared to controls (Fig 4), consistent with previous findings with 1-triple TTA [25]. However, Mildronate monotherapy also lowered acetyl-CoA carboxylase (i.e. the rate-limiting step in lipogenesis) activity without any changes in liver TAG concentrations. This suggests that lipogenesis might not be crucial for the TAG-lowering effects by 1-triple TTA. Secondly, we have previously found that omega-3 fatty acid treatment regulate plasma TAG by increasing mitochondrial fatty acid oxidation and reducing TAG synthesis through decreased DGAT activity [59]. In the present study, gene expression of ER-localized DGAT1 [60] was increased while DGAT2, co-localized to ER, mitochondria and lipid droplets [60–63] was decreased. Noteworthy, DGAT1 may be of less importance in TAG synthesis, as it cannot compensate for loss of DGAT2 [64]. As a positive correlation between TAG levels and *Dgat2* mRNA was observed, attenuated TAG biosynthesis could be partly responsible for the reduced plasma and liver TAG levels. Thirdly, downregulation of *ApoB*, *ApoCII* and *ApoCIII* and upregulation of *Vldlr* by 1-triple TTA treatment argues for a reduction in secretion of TAG and possibly an increased reuptake of VLDL particles, thus contributing to the lowering of TAG in plasma.

Mildronate has previously been shown to increase TAG levels in liver, which was not observed in this study. It is possible that a longer study period or adult rats could have been needed to reproduce this effect, as this study was conducted on 5 weeks old rats for three weeks. However, Mildronate has been used to induce fatty liver in young rats for similar and shorter time periods [22, 65]. Whether the use of a daily oral dose instead of continuously providing Mildronate in the feed is the reason for the lack of increase in hepatic TAG and OCTN2 gene expression should be considered.

In summary, carnitine biosynthesis was impaired by Mildronate, but plasma carnitine levels were not further reduced by 1-triple TTA. 1-triple TTA demonstrated lowering of plasma and hepatic TAG, despite an impaired carnitine biosynthesis, indicating efficient usage of carnitine. The TAG-lowering effect was primarily mediated by increased hepatic fatty acid oxidation, associated with mitochondrial activity, but also possibly involving reduced TAG biosynthesis and lipoprotein synthesis. These findings indicate that 1-triple TTA is a potent tool for studying lipid metabolism and mitochondrial function, and a possible candidate for treating metabolic disturbances such as fatty liver disease and carnitine deficiency.
